# The WHO-INTEGRATE evidence to decision framework version 1.0: integrating WHO norms and values and a complexity perspective

**DOI:** 10.1136/bmjgh-2018-000844

**Published:** 2019-01-25

**Authors:** Eva A Rehfuess, Jan M Stratil, Inger B Scheel, Anayda Portela, Susan L Norris, Rob Baltussen

**Affiliations:** 1 Institute for Medical Information Processing, Biometry and Epidemiology, Pettenkofer School of Public Health, LMU Munich, Munich, Germany; 2 Department of Global Health, Norwegian Institute of Public Health, Oslo, Norway; 3 Department of Maternal, Newborn, Child and Adolescent Health, World Health Organization, Geneva, Switzerland; 4 Department of Information, Evidence and Research, World Health Organization, Geneva, Switzerland; 5 Department for Health Evidence, Radboud University Medical Center, Nijmegen, The Netherlands

**Keywords:** public health, health systems, health policy

## Abstract

**Introduction:**

Evidence-to-decision (EtD) frameworks intend to ensure that all criteria of relevance to a health decision are systematically considered. This paper, part of a series commissioned by the WHO, reports on the development of an EtD framework that is rooted in WHO norms and values, reflective of the changing global health landscape, and suitable for a range of interventions and complexity features. We also sought to assess the value of this framework to decision-makers at global and national levels, and to facilitate uptake through suggestions on how to prioritise criteria and methods to collect evidence.

**Methods:**

In an iterative, principles-based approach, we developed the framework structure from WHO norms and values. Preliminary criteria were derived from key documents and supplemented with comprehensive subcriteria obtained through an overview of systematic reviews of criteria employed in health decision-making. We assessed to what extent the framework can accommodate features of complexity, and conducted key informant interviews among WHO guideline developers. Suggestions on methods were drawn from the literature and expert consultation.

**Results:**

The new WHO-INTEGRATE (INTEGRATe Evidence) framework comprises six substantive criteria—*balance of health benefits and harms*, *human rights and sociocultural acceptability*, *health equity*, *equality and non-discrimination*, *societal implications*, *financial and economic considerations*, and *feasibility and health system considerations*—and the meta-criterion *quality of evidence*. It is intended to facilitate a structured process of reflection and discussion in a problem-specific and context-specific manner from the start of a guideline development or other health decision-making process. For each criterion, the framework offers a definition, subcriteria and example questions; it also suggests relevant primary research and evidence synthesis methods and approaches to assessing quality of evidence.

**Conclusion:**

The framework is deliberately labelled version 1.0. We expect further modifications based on focus group discussions in four countries, example applications and input across concerned disciplines.

**Key questions**

**What is already known?**

- Evidence-to-decision (EtD) frameworks help to ensure that all criteria of relevance in a given guideline development or other health decision-making process are considered in a systematic way.

**What are the new findings?**

- The WHO-INTEGRATE (INTEGRATe Evidence) framework is a new EtD framework that is rooted in the norms and values of the WHO, which are agreed on by all WHO Member States.
- The framework was developed to be applicable to all health interventions, although it is particularly well suited for decisions about population-level and system-level interventions at the global as well as national levels.
- The WHO-INTEGRATE framework offers structured definitions for each of the six substantive criteria as well as the meta-criterion *quality of evidence*; example questions and suggested methods are provided to facilitate uptake.

**What do the new findings imply?**

- As part of a more holistic approach, the framework is devised as a tool to facilitate structured reflection and discussion from the beginning to the completion of a guideline development or other health decision-making process; this entails prioritisation among criteria and subcriteria to ensure appropriate evidence collection and appraisal.

## Background

Health decision-making at local, national, regional and global levels is complex,^1–3^ and can be influenced by a broad range of factors.^4–9^ Their importance varies depending on the type of health decision and the decision-making context,^10 11^ where context can relate to the institutional context (eg, Ministry of Health vs municipality), as well as the broader physical and social context, including epidemiological, geographical, sociocultural, political and other aspects.^12^ Health decision or evidence-to-decision (EtD) frameworks are intended to ensure that all important factors—in the form of decision criteria—are considered in a systematic and transparent way.^13–19^ They provide a structured approach for guideline panels or other decision-making bodies to consider the available evidence and to make informed judgements about the advantages and drawbacks of a given health decision; this approach can comprise substantive criteria as well as procedural aspects. Health decision frameworks have been applied in a variety of decision-making contexts.^20 21^

Guidelines by the WHO provide recommendations for clinical practice, public health and health system strengthening, and are intended to support health decision-makers in prioritising among or selecting suitable clinical, public health or health system interventions. When formulating recommendations, WHO generally uses an EtD framework which encompasses eight criteria: *quality of evidence* (in relation to intervention benefits and harms), *values and preferences* (in relation to outcomes), *balance of benefits and harms*, *resource implications*, *priority of the problem*, *equity and human rights*, *acceptability*, and *feasibility* (table 10.1 of the *WHO Handbook for Guideline Development*).^22^

Chapter 10 in the *WHO Handbook for Guideline Development* 22 was written by one of the lead authors of the Grading of Recommendations Assessment, Development and Evaluation (GRADE) and the GRADE EtD frameworks. The criteria in the current WHO EtD framework represent an advanced but—given their publication in 2014—not the final version of the GRADE EtD framework, which offers different versions for clinical recommendations from an individual or population perspective, coverage decisions, health system/public health decisions and recommendations about tests.^13 23^ In a recent systematic review of frameworks concerned with generic health decision-making and resource allocation processes, health technology assessments, as well as very specific health decisions, the GRADE EtD framework emerged as the best fit-for-purpose framework (Stratil *et al*, forthcoming). In particular, this framework can be applied across diverse types of health decisions and was developed following an iterative and multipronged process, combining a literature review, brainstorming and feedback from stakeholders,^24^ with application of the framework to examples and user-testing.^20 25^

However, a number of weaknesses were identified with the GRADE EtD frameworks (Stratil *et al*, forthcoming). First, the framework was developed using a pragmatic approach and lacks an explicit theoretical or conceptual basis. This makes it difficult to assess objectively whether the set of criteria is complete and organised in a meaningful way.

Second, while the frameworks are largely congruent with WHO norms and values, they do not sufficiently consider the central role of the social and economic determinants of health^26^ and the implications of health sector or intersectoral interventions for society as a whole. This is particularly important given the significant shifts in the global health landscape and the objectives and values manifest in the Sustainable Development Goals (SDGs),^27^ which are likely to shape health decision-making in the future.

A third concern is whether the decision criteria in the GRADE EtD framework are sufficiently complete and useful for decisions about complex interventions and/or the complex systems in which these are implemented, especially interventions aiming to bring about system-level changes.^28^

Fourth, the frameworks were originally developed in consultation with healthcare decision-makers in Europe, Canada and Africa, the majority of whom were physicians with significant clinical experience and research training.^24^ As a result, the frameworks may not be entirely suitable to broader public health and health system decision-making contexts, particularly in low-income and middle-income countries of Asia and Latin America.

A final and important concern relates to consistency in the application of the GRADE EtD frameworks within the WHO guideline development processes. While there are exemplar guidelines, where the WHO EtD framework has been employed as intended,^29 30^ many WHO guideline development groups focus extensively on the criterion *balance of benefits and harms* and apply the remaining criteria as a check box exercise rather than as a process that structures the development of guidelines from the start: from scoping a guideline and prioritising questions, to collecting, synthesising and appraising evidence, to formulating recommendations (SL Norris, 2017, personal communication). While there are many potential reasons for this, the current content and structure of the GRADE EtD framework may result in superficial use rather than indepth collection and assessment of evidence for the different criteria. In particular, guidance on how to frame questions for and collect evidence towards criteria beyond balance of health benefits and harms appears to be missing.

This paper, one of a series exploring the implications of complexity in systematic reviews and guideline development, reports on the development of a new EtD framework that is rooted in WHO norms and values and suitable for a broad range of health interventions, including complex interventions and interventions delivered in complex systems.

The paper addresses the following three objectives:

1. Develop an EtD framework that (a) is firmly rooted in WHO norms and values and reflective of the changing global health landscape, and (b) encompasses a comprehensive set of criteria suitable for clinical practice, public health and health system interventions.
2. Explore the value of this framework in relation to (a) complexity in individual-level as well as population-level and system-level interventions, (b) the views of developers of the WHO guidelines (global level), and (c) the views of users of the WHO guidelines (national level).
3. Facilitate uptake of the framework by emphasising the need for structured, evidence-based reflection and suggesting methods to populate the criteria with evidence in the context of decision-making under uncertainty.

The EtD framework developed out of this process is referred to as the WHO-INTEGRATE (INTEGRATe Evidence) framework version 1.0. It is proposed for use in the WHO guideline development as well as in other guideline development or health decision-making processes at the global or national level. It is intended to be used holistically—from the beginning of a health decision-making process to formulating recommendations or making a decision at the end of this process.

## Methods

**Figure 1:**
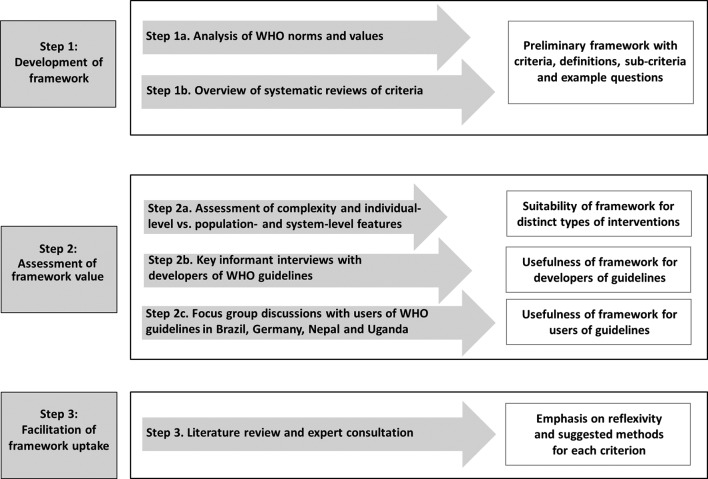
Towards a useful and operational WHO-INTEGRATE (INTEGRATe Evidence) framework.

In addressing these objectives, we followed a three-step approach, as illustrated in figure 1.

This paper provides an overview of the research project with all of its constituent components. It presents the current version of the framework (WHO-INTEGRATE framework version 1.0) and its intended use. It also reports the detailed methods and findings for steps 1a, 2a and 3, as well as an overview of the methods and findings for steps 1b and 2b. A full account of the methods and findings of step 1b is currently in preparation (Stratil *et al*, forthcoming). An integrated analysis of the views of those developing (step 2b) and using WHO guidelines (step 2c) with respect to the WHO-INTEGRATE framework will also be published separately (Stratil *et al*, forthcoming).

### Step 1: Development of the framework

*In step 1a we analysed WHO norms and values and, rooted in these norms and values, proposed a structure for the WHO-INTEGRATE framework and derived preliminary criteria.* A universally agreed normative theory for health does not exist, but most rivalling theories converge on a set of principles.^31^ As the use of these principles is less restrictive than the choice of one theory over another, we pursued a principles-based approach,^31 32^ and used WHO norms and values as the guiding principles for developing a new EtD framework. Given the complexities of normative orientation in modern pluralistic and globalised societies, we believe that WHO norms and values represent a useful foundation: they are rooted in the universally recognised concept of human rights and receive their legitimacy from having been agreed on by all 194 Member States of the WHO. To identify WHO norms and values of relevance to the process of guideline development and implementation, we used the WHO Constitution^33^ and chapter 5 *‘Incorporating equity, human rights, gender and social determinants into guidelines’* of the *WHO Handbook for Guideline Development*^22^ as a starting point. Given the emphasis in these two documents on human rights, equity and non-discrimination, social determinants of health and the role of health systems, we retrieved and analysed relevant related documents,^34–40^ including several public health ethics frameworks.^16 18 41–50^ We also reviewed the SDGs^51^ in view of their likely impact at global and national levels and as WHO is mainstreaming these throughout the organisation’s work.^52–54^

From these documents and sources, we derived principles and concepts. The structure of the WHO-INTEGRATE framework was developed via an iterative process among coauthors. We explored the meaning of different principles and concepts and assessed overlap and redundancies, making rearrangements to derive preliminary criteria. In doing so, we used a structure and wording as close as possible to the existing GRADE EtD framework to build on its strengths and to maximise potential synergies. On several occasions, we also consulted with members of the WHO Guidelines Review Committee as well as other WHO staff considered experts on selected principles or concepts (see Acknowledgements).

During the development process, we focused on substantive criteria or what decisions are based on (eg, cost, acceptability) rather than procedural criteria or how the decision-making process is conducted (eg, composition of guideline panels, participation, transparency). This is consistent with the approach promoted by the *WHO Handbook for Guideline Development*,^22^ whose overall purpose is to specify procedural rules for an objective, transparent and acceptable guideline development process. Embedded in these procedural rules, the current WHO EtD framework (table 10.1 of the *WHO Handbook for Guideline Development*)^22^—and, by extension, the WHO-INTEGRATE framework presented here—is concerned with how to facilitate the use of evidence in decision-making in a structured and comprehensive manner. It is important to note that a distinction between structural and procedural aspects is widely practised in guideline development and several other health decision-making processes,^5 6 19 55^ but is not commonly seen in the public health ethics literature.^43 44 46 56^

*In step 1b we refined the preliminary criteria derived from WHO and other related documents and supplemented them with a comprehensive set of subcriteria; we also developed definitions for criteria and example questions relating to each of the subcriteria.* We conducted an overview of systematic reviews of criteria used in decision-making, priority setting and resource allocation processes for health to derive a comprehensive set of health-relevant criteria (Stratil *et al*, forthcoming). We then compared the preliminary criteria developed in step 1a against this comprehensive set of criteria and subcriteria. To do so, one author (JMS), in a discussion with a second author (EAR), allotted the subcriteria obtained from the overview of systematic reviews to the preliminary criteria within the WHO-INTEGRATE framework. Subcriteria that did not fit were kept in a separate category. Any uncertainties were resolved in discussion with a third author (RB).

We then prepared definitions for each of the criteria using the above-described source documents for health norms and values, existing health decision frameworks (Stratil *et al*, forthcoming), and any definitions or descriptions provided in the publications included in our overview of systematic reviews of criteria (Stratil *et al*, forthcoming). Where appropriate, we also drew on additional key documents (eg, Scott *et al *57 for the definition of *acceptability*, Hultcrantz *et al *58 for the conceptualisation of *quality of evidence*, and Maeckelberghe and Schröder-Bäck^59^ for details on the subcriteria for *human rights and sociocultural acceptability* and *health equity, equality and non-discrimination*). Each definition (1) provides an overall definition of the criterion, (2) offers details and explanations regarding the subcriteria, and (3) gives guidance on how the criterion in question influences the recommendation.

As we prepared definitions, we also examined the extent to which the criteria and subcriteria relate to the intervention itself *versus* the health system and the broader context, in which an intervention is implemented. For example, the same label (eg, equity) may be employed to describe different underlying concepts, relating to *process versus outcome* (an intervention can either be implemented taking equity principles into account, or it can increase or decrease equity in health outcomes) and the *point in time* when these criteria apply (eg, equity before, during or after intervention implementation). To enable better access to sometimes abstract constructs, we also developed example questions for each of the subcriteria, drawing on the same set of documents as above.

### Step 2: assessment of framework value

*In step 2a we explored whether the WHO-INTEGRATE framework would be able to accommodate different types of health interventions and different features of complexity.* We assessed to what extent the WHO-INTEGRATE framework would be able to accommodate features of distinct types of health interventions.^60^ We broadly distinguished between interventions targeting individuals (eg, diagnosis, treatment or preventative measures addressed at individuals), interventions targeting populations, and interventions targeting the health system or other systems. Population-level interventions encompass those concerned with whole populations or population groups as defined by their age, sex, risk factor profile or other characteristics; they are often implemented in specific settings or organisations (eg, school health programmes). System-level interventions specifically redesign the context in which health-relevant behaviours occur; they are often implemented through geographical jurisdictions from national to local levels (eg, laws and regulations regarding the taxation, sale and use of tobacco products). Health system interventions represent a specific type of system-level intervention and often result in complex rearrangements across multiple health system building blocks (eg, task shifting as a process of delegating specific health service tasks from medical doctors or nurses to less specialised health workers). Interventions implemented at any of these levels can be conceptualised and analysed from a complexity perspective. To do so, we mapped core and additional components of complex interventions as defined in the iCAT_SR tool^61^ and sources of complexity in systems reported in another paper in this series^28^ against the included criteria.

*In step 2b we examined the usefulness and relevance of the WHO-INTEGRATE framework and its criteria among those developing WHO guidelines.* We conducted key informant interviews with individuals who had recently participated in a WHO guideline development process. In consultation with the Secretariat of the WHO Guidelines Review Committee, we purposively selected three ongoing or completed guidelines that had applied the current WHO EtD framework,^29 62 63^ seeking to cover distinct types of health interventions and positive as well as more difficult experiences with the application of the framework. For each guideline, we interviewed the WHO staff coordinating the guideline, the Chair of the guideline development group and the methodologist. The interviews were semistructured and used a pretested interview guide concerned with practical considerations (eg, understandability, operationalisability), as well as an assessment of missing and redundant criteria of the WHO-INTEGRATE framework. Interviewees were also asked to reflect on the implications of the WHO-INTEGRATE framework for evidence collection and guideline formulation. Interviews were held between June and November 2017 either face-to-face at the WHO Headquarters in Geneva or by telephone (JMS). Interviews were audiotaped and transcribed; data were then analysed by two researchers (JMS and IBS) using qualitative content analysis.^64^ We employed a combination of deductive (based on the guiding research questions) and inductive approaches using the software MAXQDA (VERBI Software, Berlin).

### Step 3: facilitation of framework uptake

*We critically examined how to enable use of the WHO-INTEGRATE framework as intended, and generated a table linking the criteria with suggested methods for primary research, evidence synthesis and assessing quality of evidence.* The current WHO EtD framework is intended to be used right from the planning stages of a guideline, to help derive relevant questions and structure the process, but in practice it is usually used at the end of a guideline process to help decide on the recommendations. To determine how the new framework could be used more holistically, we reflected on the literature reviewed in the context of developing the WHO-INTEGRATE framework and sought feedback from a large number of experts (see Acknowledgements). We specifically sought suggestions on how to use the framework during the early stages of the guideline development process and in a context-specific manner.

To make it easier for guideline panels to populate the criteria in the framework with evidence, we identified types of primary research, evidence synthesis methods and methods for assessing evidence quality that could inform each criterion. To accomplish this, two researchers (AP and EAR) reviewed the research questions and methods described or mentioned in the systematic review of health decision frameworks and the overview of systematic reviews (Stratil *et al*, forthcoming). We also consulted a broad range of experts comprising other authors of papers in this series, selected guideline development organisations (eg, Guidelines International Network, UK National Institute for Health and Care Excellence) and researchers with an interest in evaluating complex health technologies (see Acknowledgements).

## Results

### Developing the preliminary framework

**Figure 2:**
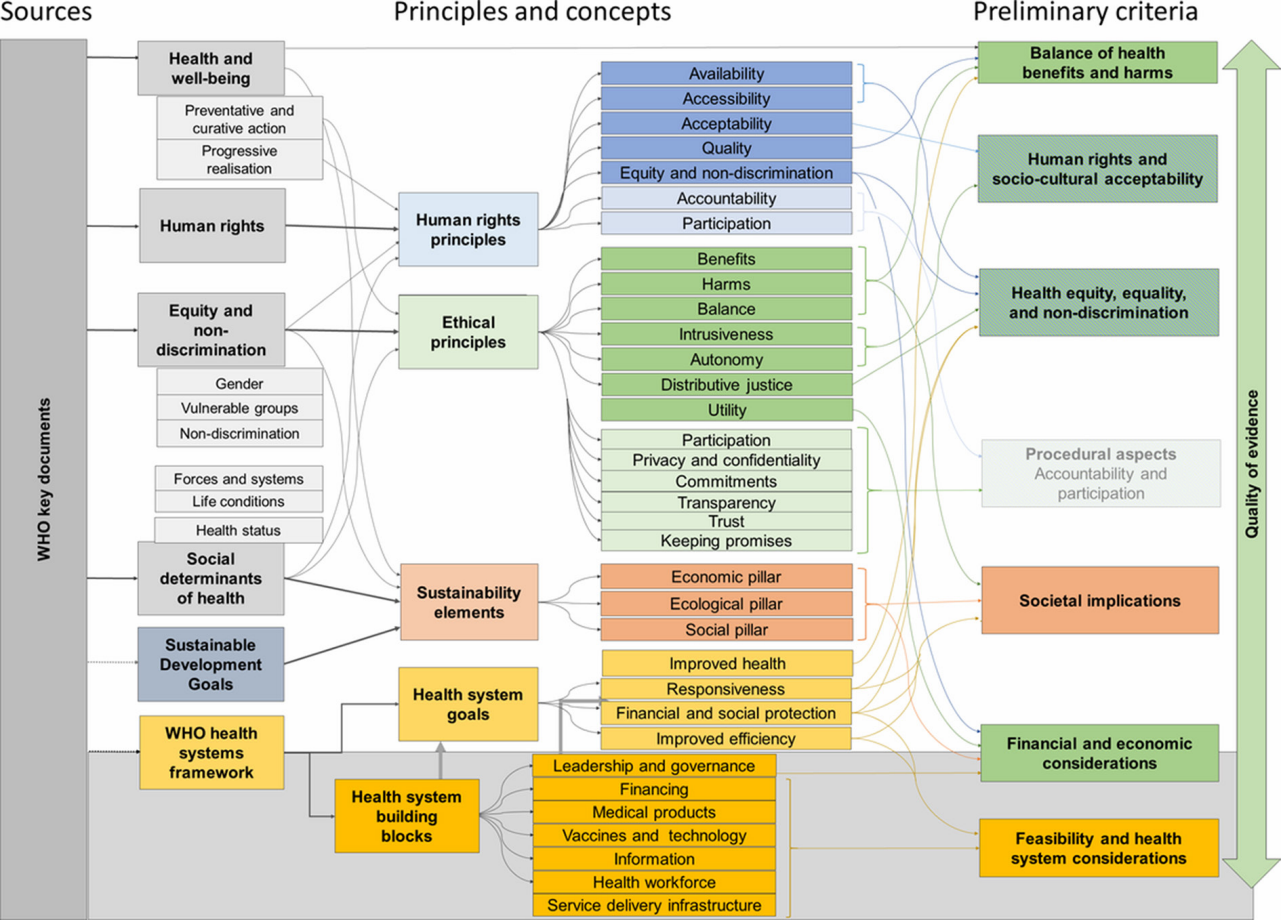
Sources and concepts for deriving principles-based preliminary criteria rooted in WHO norms and values.

Using the review of the WHO Constitution,^33^ chapter 5 of the *WHO Handbook for Guideline Development*^22^ and other source documents, we identified six major, partly overlapping concepts. Further sorting of these yielded four sets of principles and concepts (human rights principles, ethical principles, sustainability elements and health system goals and building blocks). Figure 2 illustrates how we derived preliminary criteria from WHO norms and values.

- *Human rights principles*, for the purposes of this framework, were primarily derived from international human rights law and its interpretation by the Committee on Economic, Social and Cultural Rights’ General Comment on the Right to the Highest Attainable Standard of Health (Art 12).^34^ These contain the interrelated concepts of availability and accessibility of public health and healthcare facilities, goods and services, which are required to be of appropriate quality and acceptable to users. They also include the general human rights principles of equity and non-discrimination, accountability and participation.
- Given the large number of biomedical and public health ethics frameworks,^44 46 56^ in consultation with WHO, we structured the *ethical principles* primarily according to the public health ethics framework of Childress and colleagues. This framework inter alia includes the aspects of producing benefits, avoiding harms, maximising the balance between benefits and harms, as well as distributive justice and autonomy.^41^ Based on analytical tools by the Nuffield Council of Bioethics,^45^ we also added the principle of low intrusiveness, which is related to privacy and dignity.
- Acknowledging the importance of the social determinants of health and the SDGs, we derived *sustainability elements* to capture the wide range of factors that promote conditions in which people can lead a healthy life and allow societies and individuals to develop and flourish; these sustainability elements also reflect the societal impact that interventions can have beyond health outcomes. Importantly, good health is both a precondition for achieving sustainable development and an outcome of sustainable development.^65^
- To capture the importance of feasibility of implementation as well as the impact of interventions on the health system, we used the WHO health systems framework with its four *goals* (ie, improved health, responsiveness, social and financial protection, improved efficiency) and six *building blocks* (ie, leadership and governance, financing, medical products, vaccines and technologies, information, health workforce, service delivery infrastructure).^19 38–40^

**Figure 3:**
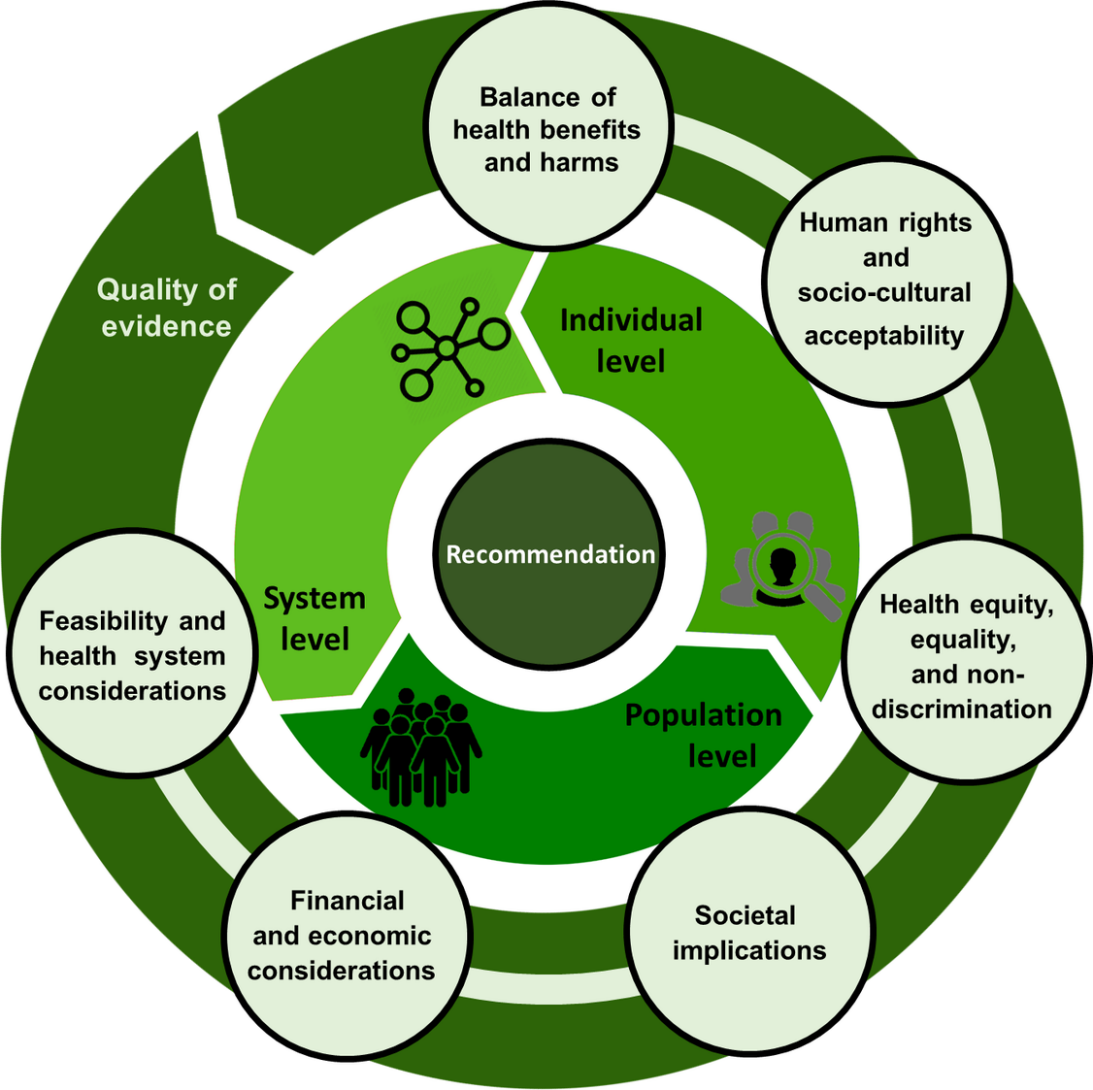
The WHO-INTEGRATE (INTEGRATe Evidence) framework version 1.0.

Figure 3 presents the WHO-INTEGRATE framework with its six criteria: *balance of health benefits and harms*, *human rights and sociocultural acceptability*, *health equity*, *equality and non-discrimination*, *societal implications*, *financial and economic considerations*, and *feasibility and health system considerations*. A seventh criterion, *quality of evidence*, represents a metacriterion that applies to each of the six substantive criteria. All seven criteria are relevant to health decision-making and the formulation of recommendations as part of the guideline development process. Each criterion may apply to interventions targeting individuals, populations or systems, or any combination of these levels.

While priority of the problem featured in both the health decision frameworks included in our systematic review (eg, Alonso-Coello *et al *13) and in the overview of systematic reviews of criteria (eg, Guindo *et al *6), we did not include this as a stand-alone substantive criterion for two reasons: First, many of the aspects included, for example, political will or public concern, are used to inform the decision to develop a guideline (or make another health decision) and thus apply before the start of the guideline development process. Second, selected aspects are captured under the other six substantive criteria, for example, burden of disease features under *balance of health benefits and harms*, and large cost of disease to health system features under *financial and economic considerations*.

### Defining criteria, subcriteria and example questions

Our overview of systematic reviews yielded more than 30 systematic reviews that contained several thousand criteria and subcriteria currently used in decision-making (Stratil *et al*, forthcoming). Recurrent aspects addressed by the subcriteria focused on the health outcomes and benefits of the intervention, health benefit for individuals and the benefit for society as a whole, the societal importance of the disease, economic considerations, quality or uncertainty of evidence, as well as population priorities, priorities within the health system and stakeholders’ interests and pressures. Feasibility criteria were concerned with the available budget, the capacities within the health system, technological complexity and acceptability of the intervention within society. Some systematic reviews were primarily concerned with interventions that would benefit vulnerable or marginalised populations (eg, children, mothers, people with lower socioeconomic status). In many reviews, normative criteria such as ethics, justice or fairness were mentioned without clear definitions or contextualisation. This comprehensive list did not yield any further criteria beyond the seven presented in figure 2. It did, however, provide many subcriteria as well as elements used in the development of detailed definitions and example questions for each criterion.

### Suitability of the framework for decisions about complex health interventions

An earlier paper in this series^28^ emphasises the importance and added value of reviewing evidence from a complex systems perspective. In developing the new EtD framework, we wanted to ensure that it would be fit for purpose when making decisions about complex interventions implemented in complex systems. We first explored to what extent different features of intervention and system complexity apply to two broad categories of interventions, that is, individual-level versus population-level and system-level interventions (table 1). Notably, even population-level and system-level interventions (eg, regulations and programmes to increase access to improved sanitation) eventually bring about changes in individual behaviour (eg, use and maintenance of toilets or latrines). Some criteria apply to a greater extent with population-level and system-level interventions (eg, *societal implications*) than individual-level interventions. Some subcriteria may take on a different meaning when applied to individual-level versus population-level and system-level interventions (eg, *autonomy*). Broadly speaking, most features of complex interventions apply to both individual-level and population-level/system-level interventions but are more salient for the latter. In contrast, many features of complex systems only apply to population-level and system-level interventions.

The last column of table 1 illustrates that distinct features of complexity do not neatly map onto specific criteria. Instead, distinct features of complexity usually affect multiple, sometimes all, criteria in the WHO-INTEGRATE framework. For example, the worked example of childhood obesity, introduced in an earlier paper in this series,^28^ discussed adaptivity of the system in response to raised taxes on soft drinks (eg, creation of lower-sugar alternatives by the soft drinks industry). This adaptivity can thus influence the *balance of health benefits and harms* (eg, consumption patterns of soft drinks change but in less pronounced ways, thereby dampening the expected effect on childhood obesity), and it may even have unwanted social consequences by stigmatising those unable to afford soft drinks (*social impact*). Raising taxes on only one sugar-sweetened product may lead to increasing the sugar content of other sugar-sweetened products (*impact on economy*, *broader positive or negative health-related impacts*) or have implications on agricultural production patterns nationally and internationally (*impact on economy and environmental impact*), illustrating the complexity of downstream implications of a ‘simple’ intervention. Drawing on the same worked example, box 1 illustrates how a simple linear perspective on the effect of an intervention will place the emphasis on one or a few criteria for decision-making, whereas a complexity perspective may take all criteria into account when making a recommendation.

Box 1 **Thinking through the criteria in relation to raised taxes on soft drinks and their implications**

A simple perspective on raising taxes on soft drinks would emphasise the linear impacts of this intervention on consumption of sugar-sweetened beverages (intermediate outcome) and different measures of childhood obesity (ultimate outcome of interest); with this perspective, the criterion *balance of health benefits and harms* would warrant the most attention. A complexity perspective on the same intervention would not start off with a preconception about a single criterion being most influential but carefully examine all criteria. For illustration purposes, this complexity perspective would examine acceptability among and likely reactions from different groups of stakeholders (eg, children, their parents), and pay specific attention to the response from vendors and producers of soft drinks (eg, potential sugar reduction in drinks with implications for the prices of these drinks), which may dampen the expected effect of the tax intervention, in terms of changes in consumption patterns, perceptions of the intervention and changes in social norms.

This complexity perspective would also encompass potential negative impacts on health equity, equality and non-discrimination (eg, expected or unexpected changes in consumption patterns across different socioeconomic or other population groups), explore positive or negative social, environmental or economic impacts (eg, changes in social norms in relation to sugar-sweetened beverages or their alternatives being more or less desirable among different population groups, changes in acceptability of further interventions to reduce sugar consumption), adopt a societal perspective in estimating the financial and economic impacts of the intervention (eg, including how costs and benefits of the raised taxes are distributed among different stakeholders and sectors), and pay attention to feasibility and health system considerations (eg, implications for human resources involved with other ongoing efforts to reduce consumption of sugar-sweetened beverages and childhood obesity).

### Usefulness of the framework from the perspective of WHO guideline developers

The key informants we interviewed had been involved in developing three very different guidelines—the WHO recommendations on antenatal care,^29^ the WHO consolidated guideline on sexual and reproductive health and rights of women living with HIV,^62^ and the WHO guideline on risk communication (online supplementary table S1).^63^ Each of these guidelines faced different challenges in terms of scope, availability of evidence and ability to incorporate multiple perspectives. All three had used the current WHO EtD framework with varying success. The diverse experiences and viewpoints of the key informants on the practical application of these criteria in guideline development were helpful in refining the framework. Further detail on and complete findings from the key informant interviews will be reported separately (Stratil *et al*, forthcoming).

10.1136/bmjgh-2018-000844.supp1 Supplementary file 1

Most participants commented positively on the WHO-INTEGRATE framework and highlighted the value of a criterion assessing societal implications, as well as the broader and more detailed specification of the criteria *human rights and sociocultural acceptability* and *health equity, equality and non-discrimination*. Two participants questioned the added value of the new EtD framework, since any guideline development process led by an experienced methodologist would automatically address the details covered in the subcriteria. Several participants were concerned about the workload that the use of the WHO-INTEGRATE framework might add to the guideline development process.

Specific remarks were made in regard to (1) missing criteria and subcriteria; (2) the hierarchy and order of criteria and subcriteria; (3) overlap and redundancies between criteria and subcriteria; (4) the precise wording and definitions of criteria; (5) the need for (more) guidance on how to use and interpret criteria and subcriteria; (6) the challenges of identifying and synthesising the required evidence; (7) resource, time and skill implications for the guideline development process; as well as (8) procedural aspects for using the framework in the guideline development process.

In response to these concerns and suggestions, we made several modifications, including changing the name and definition of several criteria and subcriteria to improve clarity and reduce overlap. We also expanded the example questions for the subcriteria to improve understandability and facilitate the development of specific questions for a given guideline. Moreover, we added suggestions on how to prioritise among criteria and subcriteria in a problem-specific and context-specific manner. Finally, we emphasised the importance of incorporating the voices of those directly affected by the recommendations into the guideline development process.

Table 2 presents the WHO-INTEGRATE framework version 1.0 criteria with abbreviated definitions and lists subcriteria. Online supplementary table S2 provides detailed definitions of the criteria as well as example questions for each of the sub criteria.

**Table 1:** Features of complex interventions (adapted from Lewin *et al*^61^) and complex systems (adapted from Petticrew *et al*^28^) and their impact on individual-level versus population-level and system-level interventions, as well as criteria in the WHO-INTEGRATE framework

Population-level and system-level interventions			Complexity-relevant differences between individual-level and population-level /system-level interventions	WHO-INTEGRATE framework criteria that are typically relevant*
Individual-level interventions				
**Features of complex interventions**				
Number of active components in the intervention^61^; interactions between components of complex interventions.^28^	+	++	Both types of interventions can comprise multiple components entailing synergistic or dissynergistic interactions among them. For population-level and system-level interventions, these interactions tend to occur among a greater number of more diverse components located at one or several organisational levels.	Balance of health benefits and harms. Human rights and sociocultural acceptability. Health inequity, equality and non-discrimination. Societal implications. Financial and economic considerations. Feasibility and health system considerations.
Number of behaviours of recipients to which the intervention is directed.	+	++	Both types of interventions can require behaviour change among recipients. For curative and preventative interventions at the individual level, these mostly relate to treatment adherence or tightly defined health-relevant behaviours, often among an ‘activated’ population seeking care or willing to engage in other ways. Population-level and system-level interventions tend to be concerned with a larger set of behaviours directly or indirectly linked to health, often in healthy general or at-risk populations.	Feasibility and health system considerations.
Range and number of organisational levels targeted by the intervention.	−	++	Individual-level interventions tend to target their recipients in a defined setting, for example, in a household or healthcare setting. Many population-level and system-level interventions target multiple levels, for example individuals living in households located in communities and influenced by community-level or national-level interventions; importantly, they often concern sectors beyond health.	Balance of health benefits and harms. Financial and economic considerations. Feasibility and health system considerations.
Level of skill required by those delivering the intervention.	++	++	The skills required for effective intervention delivery vary greatly depending on the nature of an intervention, and can be equally high for individual-level and population-level/system-level interventions. For population-level and system-level interventions, there may be a greater number of distinct implementation agents with a more diverse set of necessary skills.	Human rights and sociocultural acceptability. Feasibility and health system considerations.
Level of skill required by those receiving the intervention.	++	++	Both types of interventions can require a high level of skill among recipients, where skill can refer to specific (technical) abilities, as well as broader resources and characteristics, such as motivation and capacity (time, money, physical and mental energy). Interventions directed at individuals tend to require greater recipient skills and resources than many population-level and system-level interventions. Population-level and system-level interventions, on the other hand, often impact multiple behaviours related to diverse aspects of life and thus potentially rely on a more diverse set of skills and resources.	Human rights and sociocultural acceptability. Health inequity, equality and non-discrimination. Feasibility and health system considerations.
**Features of complex systems**				
Interactions of interventions with context and adaptation^28^/degree of tailoring intended or flexibility permitted across sites or individuals in applying or implementing the intervention.^61^	+	++	Individual-level interventions tend to involve a small degree of tailoring, typically revolving around the health professional–patient relationship. In contrast, many population-level and system-level interventions are highly context-dependent and, in order to be effective, their design and delivery strategies must be tailored to the setting or context in which they are to be implemented.	Balance of health benefits and harms. Human rights and sociocultural acceptability. Health inequity, equality and non-discrimination. Societal implications. Feasibility and health system considerations.
System adaptivity (how does the system change).	−	++	Some population-level and system-level interventions may directly attempt to change or indirectly influence the context in which they are implemented. The system thus reacts and adapts in expected or unexpected ways to the intervention.	Balance of health benefits and harms. Societal implications. Feasibility and health system considerations.
Emergent properties.	−	++	Population-level and system-level interventions tend to impact diverse aspects of life and may produce emergent features in relation to one or several of these (eg, changes in social norms). Some individual-level interventions, when implemented and viewed at the population/system level, can yield emergent features (eg, herd immunity as a result of vaccination).	Balance of health benefits and harms. Human rights and sociocultural acceptability. Health inequity, equality and non-discrimination. Societal implications.
Non-linearity and phase changes.	−	++	Some population-level interventions may only begin to deliver meaningful outcomes once they have reached a certain scale (phase changes at a threshold); they may be highly effective at particular levels of coverage and less effective at others.	Balance of health benefits and harms. Human rights and sociocultural acceptability. Societal implications.
Negative and positive feedback loops.	−	++	Population-level and system-level interventions with their specific components or the set of interacting components can produce negative feedback loops and thus reduce the overall intervention effect (damping); similarly, positive feedback loops may result in an overall intervention effect that is greater than expected.	Balance of health benefits and harms. Human rights and sociocultural acceptability. Health inequity, equality and non-discrimination. Financial and economic considerations. Feasibility and health system considerations.
Multiple (health and non-health) outcomes and long complex causal pathways.	+	++	Both types of interventions can be characterised by multiple outcomes and long, complex causal pathways. Given their large number of components impacting health as well as non-health outcomes, this feature of complex systems is particularly prevalent among population-level and system-level interventions and complicated by often long lag periods. An individual-level intervention has to be sufficiently popular and impactful to diffuse through families, peers and among the broader community or nation to eventually have population-relevant impacts, whereas a population-level or system-level intervention tends to have more immediate impacts (intended and unintended).	Balance of health benefits and harms. Human rights and sociocultural acceptability. Societal implications. Financial and economic considerations.

−, indicates not relevant; +, indicates somewhat relevant; ++, indicates highly relevant.

*Each feature of a complex system tends to influence most or all criteria; here we highlight those criteria that may be of greatest relevance.

INTEGRATE, INTEGRATe Evidence.

**Table 2:** WHO-INTEGRATE framework version 1.0: criteria with abbreviated definitions, subcriteria and implications for a recommendation. All criteria are relevant for all interventions in health decision or guideline development processes. For subcriteria there should be a discussion as to which are most relevant and if or how evidence should be collected to inform these. Online supplementary table S2 provides detailed definitions of the criteria and example questions for each of the subcriteria.

Criteria and abbreviated definitions	Subcriteria	Implications for a recommendation
**Balance of health benefits and harms** The balance of health benefits and harms reflects the magnitude and types of health impact of an intervention on individuals or populations, taking into account how those affected value different health outcomes.	Efficacy or effectiveness on health of individuals. Effectiveness or impact on health of population. Patients’/beneficiaries’ values in relation to health outcomes. Safety risk profile of intervention. Broader positive or negative health-related impacts.	The greater the net health benefit associated with an intervention, the greater the likelihood of a general recommendation in favour of this intervention.
**Human rights and sociocultural acceptability** This criterion encompasses two distinct constructs: The first refers to an intervention’s compliance with universal human rights standards and other considerations laid out in international human rights law beyond the right to health (as the right to health provides the basis of other criteria and subcriteria in this framework). The second, sociocultural acceptability, is highly time-specific and context-specific and reflects the extent to which those implementing or benefiting from an intervention as well as other relevant stakeholder groups consider it to be appropriate, based on anticipated or experienced cognitive and emotional responses to the intervention.	Accordance with universal human rights standards. Sociocultural acceptability of intervention to patients/beneficiaries and those implementing the intervention. Sociocultural acceptability of intervention to the public and other relevant stakeholder groups. Impact on autonomy of concerned stakeholders. Intrusiveness of intervention.	All recommendations should be in accordance with universal human rights standards and principles. The greater the sociocultural acceptability of an intervention to all or most relevant stakeholders, the greater the likelihood of a general recommendation in favour of this intervention.
**Health equity, equality and non-discrimination** Health equity and equality reflect a concerted and sustained effort to improve health for individuals across all populations, and to reduce avoidable systematic differences in how health and its determinants are distributed. Equality is linked to the legal principle of non-discrimination, which is designed to ensure that individuals or population groups do not experience discrimination on the basis of their sex, age, ethnicity, culture or language, sexual orientation or gender identity, disability status, education, socioeconomic status, place of residence, or any other characteristics.	Impact on health equality and/or health equity. Distribution of benefits and harms of intervention. Affordability of intervention. Accessibility of intervention. Severity and/or rarity of the condition. Lack of a suitable alternative.	The greater the likelihood that the intervention increases health equity and/or equality and that it reduces discrimination against any particular group, the greater the likelihood of a general recommendation in favour of this intervention.
**Societal implications** Societal implications recognise that health interventions do not take place in isolation and may enhance or inhibit broader social, environmental or economic goals in the short or long term. It also reflects the fact that many regulatory, environmental or other population-level health interventions are directly aimed at system-level rather than individual-level changes.	Social impact. Environmental impact.	The greater the net societal benefit associated with an intervention, the greater the likelihood of a general recommendation in favour of this intervention.
**Financial and economic considerations** Financial and economic considerations acknowledge that available financial (budgetary) resources are constrained and take into account the economic impact of an intervention on the health system, government or society as a whole.	Financial impact. Impact on economy. Ratio of costs and benefits.	The more advantageous the financial and economic implications of an intervention, the greater the likelihood of a general recommendation in favour of this intervention.
**Feasibility and health system considerations** Feasibility and health system considerations recognise that the most appropriate and feasible interventions may vary significantly across different contexts, both across countries and across jurisdictions within countries. Legislation and governance, the structure of the health system and existing programmes, as well as human resources and infrastructure, should be taken into account.	Legislation. Leadership and governance. Interaction with and impact on health system. Need for, usage of and impact on health workforce and human resources. Need for, usage of and impact on infrastructure.	The greater the feasibility of an option from the perspective of all or most stakeholders, the greater the likelihood of a general recommendation in favour of the intervention. The more advantageous the implications for the health system as a whole, the greater the likelihood of a general recommendation in favour of the intervention.
**Quality of evidence** Quality of evidence, also referred to as certainty of evidence or strength of evidence, reflects the confidence that the available evidence is adequate to support a recommendation. In principle, quality of evidence can be applied across all criteria in the WHO-INTEGRATE framework. As a large number of criteria are integrated in the decision-making process, evidence is interpreted in the broadest sense and allows for relevant contributions from a variety of disciplinary approaches. Moreover, decision-making under uncertainty often involves stakeholder experience and judgement, when stronger evidence is unavailable.	–	The greater the quality of the evidence across different criteria in the WHO-INTEGRATE framework, the greater the likelihood of a general recommendation.

INTEGRATE, INTEGRATe Evidence.

### Facilitating uptake: using the framework holistically and populating the criteria with evidence

The WHO-INTEGRATE framework is intended to improve transparency in health decision-making by supporting a structured process of reflection and discussion in a problem-specific and context-specific manner. To be most effective, this process must begin at the start of a guideline or other health decision-making process and must take evidence into account. The WHO-INTEGRATE framework is *not* intended as a ‘tick-box exercise’; there must be prioritisation of the most relevant criteria and subcriteria depending on the questions addressed by a given guideline, and the time and resources at disposition. It would be impossible and probably unnecessary for every guideline development or health decision-making process to examine all subcriteria. This flexibility can, however, lead to misuse, as stakeholders may disproportionately (eg, academics from high-income countries) or unduly (eg, participants with substantial declared or undeclared financial or other conflicts of interest) influence the decision-making process. Safeguards can be put in place through explicit procedures, in particular in relation to the composition of guideline panels or other decision-making groups. The WHO-INTEGRATE framework is also *not* an algorithm for integrating evidence across different criteria: making decisions under uncertainty and agreeing on trade-offs across criteria and subcriteria and among (and within) diverse stakeholder groups remain a core task for a guideline panel.

All criteria are important and should be reflected on, but their relevance varies depending on the type of health decision and the decision-making context. In contrast, not all subcriteria are always relevant. At the start of a guideline or other decision-making process, an appropriately composed guideline panel or other decision-making group needs to discuss which of the subcriteria are applicable and useful in relation to the nature and specific characteristics of the intervention (see table 1); this group will also need to consider the specific information needed to populate criteria or subcriteria (see table 3). Complexity in the intervention and complexity in the system into which this intervention is implemented can usually be detected; the critical question is whether it is of value to examine this complexity in depth (see box 1 in this paper and box 2 in an earlier paper in this series^28^). This prioritisation process should take the views of relevant stakeholder groups into account; which stakeholder groups are relevant depends on the nature of the problem and the institutional as well as broader physical and social context. In principle, these should include those directly affected by the intervention (eg, patients, beneficiaries), those financing (eg, health insurance providers, ministries of health, other ministries) or implementing the intervention (eg, healthcare providers, public health professionals, professionals outside of the health sector), as well as the general public.

**Table 3:** WHO-INTEGRATE framework version 1.0: criteria and suggested types of primary studies, evidence synthesis methods and approaches to assessing quality of evidence

Criteria	Types of primary studies*	Evidence synthesis or mapping methods	Pragmatic approaches	Approaches to assessing quality of evidence
Balance of health benefits and harms.	Efficacy or effectiveness on health of individuals/populations: RCTs, pragmatic trials, quasi-experimental studies, comparative observational studies; longer term observational studies, modelling (eg, transmission modelling for infectious diseases). Patients’/beneficiaries’ values in relation to health outcomes: qualitative studies (eg, semistructured interviews, focus groups), cross-sectional studies. Safety risk profile of intervention: RCTs, quasi-experimental studies, comparative observational studies for anticipated harms; registry studies, longer term observational studies, case series, case reports for unanticipated effects. Broader positive or negative health-related impacts: RCTs, quasi-experimental studies, observational studies, qualitative studies.	Systematic reviews of efficacy/effectiveness^83^ for anticipated effects. Qualitative evidence syntheses^84 85^ and mixed-method reviews^86^ or cross-sectional studies^66^ for patients’/beneficiaries’ values in relation to health outcomes. Scoping reviews^87 88^ for unanticipated effects.	Rapid reviews of efficacy/effectiveness.^89–91^ Overviews of systematic reviews.^83 92^	GRADE.^70 71 73^
Human rights and sociocultural acceptability.	Accordance with universal human rights standards: mapping of relevant aspects, pro et contra analysis,^93^ ethical analysis (eg, casuistry, coherence analysis, wide reflective equilibrium),^94^ power analyses, human rights impact assessment.^95^ Sociocultural acceptability of intervention, impact on autonomy of concerned stakeholders, intrusiveness of intervention: mapping of relevant aspects, pro et contra analysis,^93^ discourse analysis, qualitative studies (ideally longitudinal to examine changes over time), discrete choice experiments, cross-sectional studies,^66^ longitudinal quantitative studies (to examine changes over time), mixed-method studies.	Ethics syntheses^96 97^ for accordance with universal human rights standards. Qualitative evidence syntheses^84 85 98^ and mixed-method reviews^86^ for sociocultural acceptability and impact on autonomy of concerned stakeholders and intrusiveness of interventions.	Purposively selected studies from different contexts (to illustrate broad spectrum of issues).	GRADE CERQual^72 99^ (where applicable). Q-SEA for ethics analyses.^57^
Societal implications.	Social impacts: RCTs, quasi-experimental studies, comparative observational studies, longitudinal implementation studies, qualitative studies, case studies, power analyses. Environmental impacts: RCTs, quasi-experimental studies, comparative observational studies, longitudinal implementation studies, qualitative studies, case studies, environmental impact assessments, modelling studies. Combined social, environmental and economic impacts: health impact assessments, modelling studies (eg, decision-analytical modelling).	Systematic reviews of effectiveness.^83^ Qualitative evidence syntheses.^11 84 85^ Mixed-method reviews.^86^ Health technology assessments.^68^	Purposively selected studies from different contexts (to illustrate broad spectrum of issues).	No standardised approach. GRADE^70 71^ (where applicable).
Health equity, equality and non-discrimination.	Impact on health equality and/or health equity, distribution of benefits and harms of intervention: human rights impact assessment,^95^ disaggregated RCTs, quasi-experimental or comparative observational studies, RCTs and quasi-experimental or comparative observational studies conducted in disadvantaged groups,^100^ power analyses, GIS-based studies, qualitative studies, ethical analysis. Affordability of intervention: cross-sectional or longitudinal observational studies, discrete choice experiments, qualitative studies, catastrophic health expenditure studies. Accessibility of intervention: health system barrier studies, cross-sectional or longitudinal observational studies, discrete choice experiments, qualitative studies, ethical analysis, GIS-based studies. Severity and/or rarity of the condition: health state valuations, cross-sectional studies for severity of condition; observational studies for frequency (incidence, prevalence) of condition. Lack of a suitable alternative: situation analysis of intervention options; quantitative or qualitative studies of adverse effects of existing options.	Quantitative systematic reviews^83^ using PROGRESS^101^ or PROGRESS PLUS,^102^ where possible using prespecified subgroup analyses. Quantitative systematic reviews targeting disadvantaged groups. Equity weights and social welfare functions in economic analyses (*see Financial and economic considerations*). Qualitative evidence syntheses^11 84 85^ and mixed-method reviews.^86^ Ethics syntheses.^96 97^	Purposively selected studies from different contexts (to illustrate broad spectrum of issues). Scoping reviews.^87 88^ Overviews of systematic reviews.^83 92^	No standardised approach. GRADE^70 71^ for subgroup analyses (where applicable). Relevant considerations, such as including health equity as an outcome, in Welch *et al.*^103^
Financial and economic considerations.	Financial impact: prices and price justifications for unit costs per beneficiary/population according to relevant perspectives, budget impact analysis.^104^ Impact on economy: economic burden of disease studies,^105^ quasi-experimental studies, comparative observational studies, longitudinal implementation studies, qualitative studies, case studies, modelling studies. Ratio of costs and benefits: economic analyses as a comparative analysis of alternative courses of action in terms of their costs and consequences (eg, cost-minimisation analysis, cost-effectiveness analysis, cost-utility analysis, cost-benefit analysis).	Comprehensive or representative cost or budget impact data at the appropriate level (global, regional, national, subnational). Economic burden of disease studies undertaken at the appropriate level (global, regional, national, subnational). Economic analyses undertaken at the appropriate level^106 107^ or economic analysis reviews.^108–111^	Cost or budget impact data for purposively selected contexts. Economic analyses undertaken for selected contexts.	No standardised approach. Relevant considerations in Drummond *et al*^106^ (chapter 3 and box 3.1) and Brunetti *et al*.^112^
Feasibility and health system considerations.	Legislation, leadership and governance, interaction with and impact on health system, need for, usage of and impact on health workforce, human resources and infrastructure: health systems research,^113^ including mapping of relevant aspects, situation analysis, cross-sectional studies, qualitative studies, case studies.	Qualitative evidence syntheses,^11 84 85^ mixed-method reviews.^86^	Formal consultation of content experts.	No standardised approach. GRADE CERQual^72^ (where applicable).

*This table offers a collection of suitable methods rather than guidance on the most appropriate method, which depends on the specific research question. Where appropriate, the order in which the methods are presented implies a hierarchy of evidence (eg, RCTs are more suited to assessing questions of efficacy than modelling).

GIS, geographical information system; GRADE, Grading of Recommendations Assessment, Development and Evaluation; GRADE CERQual, Confidence in the Evidence from Reviews of Qualitative Research; HTA, health technology assessment; INTEGRATE, INTEGRATe Evidence; Q-SEA, Quality Standards for Ethics Analyses in HTA; RCT, randomised controlled trial.

A systematic weakness in many guideline development and other health decision-making processes is that consumer participation is obviated and guideline panels often substitute their own values and views for those of patients/beneficiaries. The voices of patients/beneficiaries and other relevant stakeholder groups can be incorporated through direct participation or representative surveys^66^ as well as qualitative research (see table 3).

The guideline panel will also need to decide how best to populate the criteria with evidence and whether a formal evidence synthesis or a more pragmatic approach is warranted for each. This decision will be influenced by the relevance of criteria and subcriteria in relation to a specific intervention or decision, and by the likely types and quantity of evidence available, as well as time and resource constraints. At the end of the process, the guideline panel will need to reassess the criteria and relevant subcriteria in light of the assembled evidence and make a judgement regarding each criterion.

Table 3 suggests relevant types of primary research, evidence synthesis or mapping methods, streamlined or pragmatic approaches, as well as methods to assess the quality of evidence for each of the six substantive criteria. We provide a collection of suitable primary research and synthesis approaches, but make no firm distinction between more or less suitable methods. We note that the approach to gathering evidence may depend on the criterion: for some criteria a systematic review will be most appropriate, while for others a representative survey or other single primary study may be more suitable. Surprisingly, the majority of the health decision frameworks included in our systematic review (Stratil *et al*, forthcoming) did not offer insights for operationalising frameworks, for example by specifying research questions or suggesting methods for primary research or evidence synthesis. The GRADE EtD framework^13 67^ and the EUnetHTA (EUropean network for Health Technology Assessment) core model^68^ provided some methods. We also identified relevant information in the following sources: the EVIDEM (Evidence and Value: Impact on DEcisionMaking) framework,^14^ Marckmann and colleagues,^16^ the health systems framework^19^ and publications included in our overview of systematic reviews of criteria.^69^ Expert consultation played a critical role in identifying methods for inclusion in table 3.

## Discussion

### Added value of the WHO-INTEGRATE framework

The WHO-INTEGRATE framework represents a new comprehensive EtD framework that is rooted in WHO norms and values. It offers an explicit conceptualisation of each criterion and a rationale for including relevant concepts as criteria or subcriteria. The WHO norms and values apply across all WHO Member States and settings, and the new framework should, in principle, be relevant for health decision-making at global, national and subnational levels. It reflects a broad understanding of health and its determinants and takes account of complex interventions and complex systems perspectives. It emphasises sustainability and the interconnectedness between health and other sectors, inherent in the SDGs. While the framework is conceived for individual-level, population-level and system-level interventions, it is likely to be particularly well suited for public health and health system interventions characterised by complexity and/or approached from a complexity perspective. The WHO-INTEGRATE framework is intended as a tool to facilitate structured reflection and discussions from the beginning of a guideline development or other health decision-making process. This has ramifications in terms of the need to prioritise among criteria and subcriteria and the need to collect evidence for each. The framework supports this process by offering structured definitions for each criterion and example questions for each subcriterion, and by suggesting methods for primary research, evidence synthesis and assessing the quality of the evidence.

There are many similarities between the WHO-INTEGRATE framework and the widely used GRADE EtD framework. As stated in our methods, we deliberately attempted to stay as close as possible to the GRADE EtD framework, thus building on established terms and concepts (eg, *balance of health benefits and harms*). In contrast, criteria with a strong normative foundation (eg, *health equity, equality and non-discrimination)* were much less developed in the GRADE EtD framework; notably, the criterion *societal implications*, which has its roots in the recognition of the multisectoral determinants of health, is absent from the GRADE EtD framework. There are also more fundamental differences. While the GRADE EtD framework emphasises the efficacy/effectiveness of interventions and their potential harmful impacts, there is no inherent weighting of criteria in the WHO-INTEGRATE framework: guideline panels must decide in a context-specific and problem-specific manner which criteria and subcriteria are most relevant. Moreover, in contrast to the narrower certainty of evidence concept in the GRADE EtD framework, the WHO-INTEGRATE framework has deliberately adopted a broad quality of evidence concept that applies across all criteria and is not linked to a prespecified grading system. For several criteria (and/or subcriteria) GRADE^70 71^ and GRADE CERQual (Confidence in the Evidence from Reviews of Qualitative Research^72^) are the most appropriate approaches to examining quality of evidence, and we would encourage users of the WHO-INTEGRATE framework to adopt these. In fact, another paper in this series explores how complexity can be considered when assessing the certainty of evidence on intervention effectiveness.^73^ For other criteria (and/or subcriteria), these existing tools are not well suited, and we hope that more appropriate approaches will become available—whether through further developments within the GRADE Working Group or independent efforts.

The GRADE EtD framework allows for tailoring of criteria, for example by considering a detailed judgement as a stand-alone criterion or by removing a criterion from the GRADE EtD framework and considering it prior to the start of the decision-making process^13^; in fact, refinement of the GRADE EtD framework continues and has already resulted in suggestions towards more detailed specifications of selected criteria.^74^ Similarly, we expect various developments towards a version 2.0 of the WHO-INTEGRATE framework (see below). We thus envisage specific innovations to be adopted across these evolving frameworks and, potentially, convergence over time.

### Strengths and limitations of the development process

In developing the WHO-INTEGRATE framework, we combined a principles-based approach with an overview of systematic reviews of decision criteria and thus ensured a solid, comprehensive normative foundation. We were explicit and transparent as to how criteria (see figure 1) and subcriteria (Stratil *et al*, forthcoming) were derived. While there is some conceptual overlap at the level of the criteria (eg, *societal implications* and *financial and economic considerations*), there are no significant redundancies among the subcriteria (Stratil *et al*, forthcoming). Cross-linkages among the criteria are emphasised in the definitions and example questions.

Solely adapting the substantive criteria may be insufficient to overcome limitations in guideline development or other decision-making processes.^22^ The WHO-INTEGRATE framework is concerned with substantive criteria; it does *not* comprise procedural criteria but is intended to be embedded in a clearly specified health decision-making process as described, for example, in the *WHO Handbook for Guideline Development*.^22^ We recognise that transparent and inclusive procedures are essential to achieve legitimate health decisions and to resolve reasonable disagreement based on competing criteria and the various individual, social, cultural and political values affecting their interpretation and the explicit or implicit weight assigned to them. In this context legitimacy refers to the reasonableness, or acceptability, of decisions as perceived by the population.^75 76^ Compromised legitimacy may hinder the effective implementation of guidelines or other health-relevant decisions. Transparent and inclusive procedures require, among other considerations, the involvement of relevant stakeholders in the decision-making process, the public announcement of forthcoming decisions including their underlying argumentation, and the instalment of mechanisms for appeal.^75 76^ This is relevant for the development of WHO guidelines at the global level, as well as their adaptation at the national or subnational levels, where a wide array of stakeholders with diverse sets of values should be involved.^77 78^ In our overview of systematic reviews, we distilled procedural criteria (Stratil *et al*, forthcoming) and suggest that these be reviewed separately to inform guideline development and other health decision processes.^18 41 42 45 75 76 79^ We also refer to evidence-informed deliberative processes, which explicitly integrate the use of substantive criteria with procedural criteria to set priorities at national and subnational levels.^80–82^

The WHO-INTEGRATE framework is a highly interdisciplinary framework: each criterion, especially those criteria that are less developed in current EtD frameworks (eg, *human rights and sociocultural acceptability*) or absent from the literature (eg, *societal implications*), merits research to unpack them and, where applicable, provide a more detailed normative justification. We anticipate constructive input from and exchange with relevant disciplines, in particular public health ethics but also sociology, environmental sciences, economics and many others. Future collaborative research is expected to lead to a WHO-INTEGRATE framework version 2.0. This may advance the criteria and subcriteria and their normative foundations, as well as methodological approaches to populate these criteria with evidence.

To examine the value of the WHO-INTEGRATE framework to potential users, we conducted empirical qualitative research. Insights from interviews with key informants in relation to their recent experiences with developing WHO guidelines led to several refinements in the wording of the criteria and subcriteria and highlighted the importance of providing example questions as well as suggested methods. We expect that the second empirical qualitative research component, focus group discussions in Nepal, Uganda, Germany and Brazil, will yield additional insights from different perspectives and possibly further modifications to the framework. An integrated analysis of the views of WHO guideline developers and users will be published separately (Stratil *et al*, forthcoming).

Several of our key informants expressed concern about the potential workload resulting from collecting evidence for each of the criteria and, in particular, for the many subcriteria in the WHO-INTEGRATE framework. Both the process of prioritisation and the process of collecting evidence—through high-quality evidence synthesis or more pragmatic approaches—need to be tested in practice. We anticipate sharing worked examples and developing additional guidance on how to implement the framework in practice.

## Conclusions

The WHO-INTEGRATE framework represents a comprehensive EtD framework rooted in WHO norms and values that is, in principle, suitable for individual-level, population-level and system-level health interventions that may or may not be characterised by complexity. It offers structured definitions for each of the six substantive criteria as well as the meta-criterion quality of evidence; example questions and suggested methods are provided to facilitate uptake. Importantly, this framework is intended to be used from the beginning and throughout a guideline or other health decision-making process, whether this process takes place at the global, national or subnational level. In working towards version 2.0, we welcome learning from the experiences of those applying the framework, as well as from researchers in disciplines concerned with the included criteria or subcriteria.
