# Supplementary material for: The WHO-INTEGRATE evidence to decision framework version 1.0: integrating WHO norms and values and a complexity perspective
Source: BMJ Glob Health. 2019 Jan 25;4(Suppl 1):e000844. doi: 10.1136/bmjgh-2018-000844 (PMC6350705; doi:10.1136/bmjgh-2018-000844)
Supplement: Supplementary file 1 [file bmjgh-2018-000844supp001.docx]

**Supplementary File**

**The WHO-INTEGRATE evidence to decision framework version 1.0: Integrating WHO norms and values and a complexity perspective**

**Eva A Rehfuess^1^*, Jan M Stratil^1^*, Inger B Scheel^2^, Anayda Portela^3^, Susan L Norris^4^, Rob Baltussen^5^**

* joint first authorship

^1^ Institute for Medical Information Processing, Biometry and Epidemiology, Pettenkofer School of Public Health, LMU Munich, Marchioninistr. 15, 81377 Munich, Germany

^2^ Department of Global Health, Norwegian Institute of Public Health, PO Box 4404, Nydalen, 0403 Oslo, Norway

^3^ Department of  Maternal, Newborn, Child and Adolescent Health, World Health Organization, Avenue Appia 20, 1211 Geneva 27, Switzerland

^4^ Department of Information, Evidence and Research, World Health Organization, Avenue Appia 20, 1211 Geneva 27, Switzerland

^5^ Department for Health Evidence, Radboud University Medical Center, P.O.Box 9101, 6500 HB Nijmegen, The Netherlands

Corresponding author:

Dr Eva A. Rehfuess

Tel: +49 (0)89 4400 77494

Fax: +49 (0)89 4400 77491

Email: [rehfuess@ibe.med.uni-muenchen.de](mailto:rehfuess@ibe.med.uni-muenchen.de)

**Table S1.** Overview of key informant interviews

| Number of interviews | 9 |
| --- | --- |
| Guideline that key informants had contributed to | - “Consolidated guideline on sexual and reproductive health and rights of women living with WHO recommendations on antenatal care for a positive pregnancy experience”: 3 - “WHO recommendations on antenatal care for a positive pregnancy experience”: 3 - “A WHO Guideline for Emergency Risk Communication (ERC) policy and practice”: 3 |
| Gender | Male: 2  Female: 7 |
| Mode of interview | Face to face: 3  By telephone or skype: 6 |
| Duration of interview | Median: 65 minutes (54 minutes; 71 minutes) |

**Table S2.** WHO-INTEGRATE framework version 1.0: criteria with detailed definitions and implications for a recommendation, sub-criteria and example questions

| **Criteria**  Definition | **Sub-criteria and example questions**  All criteria are relevant for all interventions in health decision or guideline development processes. For sub-criteria there should be a discussion as to which are most relevant and if or how evidence should be collected to inform these. | |
| --- | --- | --- |
| **Balance of health benefits and harms**  The balance of health benefits and harms reflects the magnitude and types of health impact of an intervention on individuals or populations, taking into account how those affected value different health outcomes.  Both positive and negative impacts on health must be considered, aiming to measure outcomes rather than surrogate or process markers and including those health outcomes most valued by patients/beneficiaries. Where possible, evidence of real-world effectiveness rather than efficacy under controlled circumstances should be used and a long-term perspective adopted.  The greater the net health benefit associated with an intervention, the greater the likelihood of a general recommendation in favour of this intervention. | - **Efficacy or effectiveness on health of individuals** | - What is the efficacy (under controlled, often ideal circumstances) or effectiveness (in a real-life setting) of the intervention on the health of individuals, including patient-reported outcomes? Does efficacy or effectiveness vary in the short- versus longer-term? |
|  | - **Effectiveness or impact on health of population** | - What is the effectiveness or impact of the intervention on the health of the population, including on beneficiary-reported outcomes? Can individual-level effects be aggregated at the population level, or do important system dynamics (e.g. positive or negative feedback loops) play a role? Does effectiveness or impact vary in the short- versus longer-term? |
|  | - **Patients’/beneficiaries’ values in relation to health outcomes** | - To what extent do patients/beneficiaries value different health outcomes? |
|  | - **Safety-risk-profile of intervention** | - Which adverse events are associated with the intervention, including the risk of the intervention being misused? |
|  | - **Broader positive or negative health-related impacts** | - Which broader positive or negative health-related impacts, such as impact on other diseases, over-diagnosis and spillover effects beyond patients/beneficiaries, are associated with the intervention? Are there features of the intervention that increase or reduce stigma associated with the disease and that lead to health consequences (see *Societal implications*)? |
| **Human rights and socio-cultural acceptability**  This criterion encompasses two distinct constructs: The first refers to an intervention’s compliance with universal human rights standards and other considerations laid out in international human rights law beyond the right to health (as the right to health provides the basis of other criteria and sub-criteria in this framework). The second, socio-cultural acceptability, is highly time- and context-specific and reflects the extent to which those implementing or benefiting from an intervention as well as other relevant stakeholder groups consider it to be appropriate, based on anticipated or experienced cognitive and emotional responses to the intervention.Socio-cultural acceptability is affected by socio-cultural norms and preferences as well as power dynamics in relation to sex, age, ethnicity, culture or language, sexual orientation or gender identity, disability status, education, socio-economic status, place of residence or other characteristics. It may also be affected by the different values assigned to considerations of autonomy and intrusiveness (including privacy and dignity), freedom of information and movement, as well as the distribution of benefits, harms and costs. Socio-cultural acceptability can vary greatly between stakeholder groups, and health interests should always take precedence over commercial interests. Where applicable, aspects of socio-cultural acceptability are to be assessed in comparison with usual care/the status quo or alternative interventions and should keep likely changes over time and across different population and stakeholder groups in mind.  All recommendations should be in accordance with universal human rights standards and principles.  The greater the socio-cultural acceptability of an intervention to all or most relevant stakeholders, the greater the likelihood of a general recommendation in favour of this intervention. | - **Accordance with universal human rights standards** | - Is the intervention in accordance with universal human rights standards and principles? |
|  | - **Socio-cultural acceptability of intervention to patients/ beneficiaries and those implementing the intervention** | - Is the intervention socio-culturally acceptable to patients/beneficiaries as well as to those implementing it? To which extent do patients/beneficiaries value different non-health outcomes? |
|  | - **Socio-cultural acceptability of intervention to the public and other relevant stakeholder groups** | - Is the intervention socio-culturally acceptable to the public and other relevant stakeholder groups? Is the intervention sensitive to sex, age, ethnicity, culture or language, sexual orientation or gender identity, disability status, education, socio-economic status, place of residence or any other relevant characteristics? |
|  | - **Impact on autonomy** **of concerned stakeholders** | - How does the intervention affect an individual’s, population group’s or organization’s autonomy, i.e. their ability to make a competent, informed and voluntary decision? |
|  | - **Intrusiveness of intervention** | - How intrusive is the intervention, ranging from low intrusiveness (e.g. providing information) to intermediate intrusiveness (e.g. guiding choices) to high intrusiveness (e.g. restricting or eliminating choices)? Where applicable, are high intrusiveness and/or impacts on the privacy and dignity of concerned stakeholders justified? |
| **Health equity, equality, and non-discrimination**  Health equity and equality reflect a concerted and sustained effort to improve health for individuals across all populations, and to reduce avoidable systematic differences in how health and its determinants are distributed. Equality is linked to the legal principle of non-discrimination, which is designed to ensure that individuals or population groups do not experience discrimination on the basis of their sex, age, ethnicity, culture or language, sexual orientation or gender identity, disability status, education, socio-economic status, place of residence or any other characteristic.  Firmly rooted in the right to health and international human rights law and aiming to promote social justice, this criterion is concerned with the distribution of a condition, of its determinants and of the effects of interventions across different population groups. Sometimes interventions improve health for the population as a whole (see *Balance of health benefits and harms*) but – either in the short term, or over prolonged periods of time – negatively affect the distribution of health. *Health inequalities* constitute systematic differences between population groups; *health inequities* refer to those inequalities that are avoidable and deemed unfair. Interventions may either decrease or increase such differences through their affordability (including protection against unwanted financial and social consequences of taking up services) and accessibility (which is generally described as the physical and information-based distribution of health-relevant goods and services but may also encompass non-financial investments from recipients, e.g. time, energy) or acceptability (see *Human rights and socio-cultural acceptability*). In addition, this criterion recognizes that society may (or may not) attach greater value to interventions that target severe conditions, rare diseases, or conditions for which there is no suitable alternative.  The greater the likelihood that the intervention increases health equity and/or equality and that it reduces discrimination against any particular group, the greater the likelihood of a general recommendation in favour of this intervention. | - **Impact on health equality and/or health equity** | - How are the condition and its determinants distributed across different population groups? Is the intervention likely to reduce or increase existing health inequalities and/or health inequities? Does the intervention prioritise and/or aid those furthest behind? How do such impacts on health inequalities and /or health inequities vary over time, e.g. are initial increases likely to balance out over time, as interventions are scaled up? |
|  | - **Distribution of benefits and harms of intervention** | - How are the benefits and harms of the intervention distributed across the population? Who carries the burden (e.g. all), who benefits (e.g. a very small sub-group)? |
|  | - **Affordability of intervention** | - How affordable is the intervention for individuals, households or communities? How will it impact household health expenditures, including risk of catastrophic health expenditures and health-related financial risks? |
|  | - **Accessibility of intervention** | - How accessible - in terms of physical as well as informational access - is the intervention across different population groups? |
|  | - **Severity and/or rarity of the condition** | - Does the intervention address a particularly severe condition (e.g. life-threatening, end-of-life, affecting individuals with a low pre-existing health status)? Does it address a rare condition? |
|  | - **Lack of a suitable alternative** | - Is there any suitable alternative to addressing the condition, does the intervention represent the only available option? Is this option proportionate to the need, and will it be subject to periodic review? |
| **Societal implications**  Societal implications recognize that health interventions do not take place in isolation and may enhance or inhibit broader social, environmental or economic goals in the short- or long-term. It also reflects the fact that many regulatory, environmental or other population-level health interventions are directly aimed at system-level rather than individual-level changes.  This criterion acknowledges that the right to health embraces a wide range of socio-economic and other underlying determinants of health that may or may not lead to conditions, in which people can lead a healthy life; these determinants operate across different sectors and organizational levels. The criterion also acknowledges that promoting health must go hand-in-hand with strategies to end poverty and address a broad range of social needs and to build economic growth (see *Impact on economy* under *Financial and economic considerations*), while tackling climate change and environmental protection.  The greater the net societal benefit associated with an intervention, the greater the likelihood of a general recommendation in favour of this intervention. | - **Social impact** | - What is the social impact of the intervention: Are there features of the intervention that increase or reduce stigma and that lead to social consequences (see *Balance of health benefits and harms*)? Does the intervention enhance or limit social goals, such as education, social cohesion and the attainment of various human rights beyond health? Does it change social norms at individual or population level? Does it impact research and innovation? |
|  | - **Environmental impact** | - What is the environmental impact of the intervention? Does it contribute to or limit the achievement of goals to protect the environment and efforts to mitigate or adapt to climate change? |
| **Financial and economic considerations**  Financial and economic considerations acknowledge that available financial (budgetary) resources are constrained and take into account the economic impact of an intervention on the health system, government or society as a whole.  Embedded in this criterion is the idea of progressive realization, which implies being resource-conscious in moving as expeditiously and effectively as possible towards the full realization of the right to health. The criterion also captures the notion of opportunity costs, operationalised through the cost-effectiveness or cost-benefit of an intervention – these reflect the health gains that would be foregone, if resources were spent on alternative interventions. Positive and negative financial and economic implications must be considered – from a health system or broader societal perspective, depending on the intervention concerned. Where possible and appropriate, a long-term perspective should be adopted and a formal economic evaluation conducted.  The more advantageous the financial and economic implications of an intervention, the greater the likelihood of a general recommendation in favour of this intervention. | - **Financial impact** | - What is the cost of the intervention? What is the overall budget impact of implementing the intervention? Do cost and budget impacts vary in the short- versus longer-term, and are they sustainable? |
|  | - **Impact on economy** | - What is the overall economic impact of the intervention? How are different types of economic impact distributed, how does the intervention influence different sectors at different organizational levels? Does it contribute to or limit the achievement of broader development and poverty reduction goals? How does it impact the working population, for example in terms of who participates in the workforce and their level of engagement? |
|  | - **Ratio of costs and benefits** | - What is the value-for-money of the intervention, based on an appropriate choice of method, e.g. cost-effectiveness, cost-benefit or cost-utility? |
| **Feasibility and health system considerations**  Feasibility and health system considerations recognize that the most appropriate and feasible interventions may vary significantly across different contexts, both across countries and across jurisdictions within countries. Legislation and governance, the structure of the health system and existing programmes as well as human resources and infrastructure should be taken into account.  Barriers (e.g. lack of human resources, opposing legislation) as well as facilitators (e.g. an intervention fitting with previous spending patterns and/or existing programmes) should be recognized. When considering the fit of the intervention with the health system and its likely impacts on human resources and infrastructure at various levels, a broad societal and longer-term perspective should be adopted, where appropriate.  The greater the feasibility of an option from the perspective of all or most stakeholders, the greater the likelihood of a general recommendation in favour of the intervention. The more advantageous the implications for the health system as a whole, the greater the likelihood of a general recommendation in favour of the intervention. | - **Legislation** | - Are there any legal barriers or facilitators to the implementation of the intervention? |
|  | - **Leadership and governance** | - Might governance aspects, such as past decisions and strategic considerations, positively or negatively impact the implementation of the intervention? Are formal or informal institutions available to provide effective leadership, oversight and accountability in implementing the intervention? |
|  | - **Interaction with and impact on health system** | - How does the intervention interact with the existing health system? Is it likely to fit well or not, is it likely to impact on it in positive or negative ways? |
|  | - **Need for, usage of and impact on health workforce and human resources** | - How does the intervention interact with the need for and usage of the existing health workforce and broader human resources (in the health sector or other sectors), at national and sub-national levels? Is it likely to impact on these in positive or negative ways, for example by affecting the number or distribution of staff, their skills, responsiveness or productivity? |
|  | - **Need for, usage of and impact on infrastructure** | - How does the intervention interact with the need for and usage of the existing health system infrastructure (e.g. types of health facilities, health information system, medical products and technologies) as well as other relevant infrastructure (e.g. transportation, energy), at national and sub-national levels? Is it likely to impact on these and their performance in positive or negative ways? |
| **Meta-criterion: Quality of evidence** | Quality of evidence, also referred to as certainty of evidence or strength of evidence, reflects the confidence that the available evidence is adequate to support a recommendation. In principle, quality of evidence can be applied across all criteria in the WHO-INTEGRATE framework – balance of health benefits and harms, human rights and socio-cultural acceptability, health equity, equality and non-discrimination, societal implications, financial and economic considerations and feasibility and health system considerations. As a large number of criteria are integrated in the decision-making process, evidence is interpreted in the broadest sense, and allows for relevant contributions from a variety of disciplinary approaches. Moreover, decision-making under uncertainty often involves stakeholder experience and judgement, when stronger evidence is unavailable.  In relation to effectiveness and impact, quality of evidence or certainty of evidence has variably been interpreted as confidence in (i) point estimates, (ii) the true effect lying above (or below) a certain threshold, (iii) the true effect lying within a 95% confidence interval, and (iv) the intervention being effective or not (i.e. a non-null effect). In relation to human rights and socio-cultural acceptability, quality of evidence has been described as the extent to which a finding is a reasonable representation of the phenomenon of interest. Quantity, quality (often described as internal validity) and consistency of evidence are among the most widely described underlying concepts; in addition, relevance of evidence (often referred to as external validity or generalizability) plays an important role. How quality of evidence is assessed depends on the criterion in question and the nature of a given body of evidence, e.g. GRADE is widely used for questions of effectiveness, whereas GRADE CERQual is suitable for rating qualitative evidence (**Table 3**).  The greater the quality of the evidence across different criteria in the WHO-INTEGRATE framework, the greater the likelihood of a general recommendation. | |
